# Intravaginal lactic acid gel versus oral metronidazole for treating women with recurrent bacterial vaginosis: the VITA randomised controlled trial

**DOI:** 10.1186/s12905-023-02303-5

**Published:** 2023-05-09

**Authors:** Jonathan D. C. Ross, Clare Brittain, Jocelyn Anstey Watkins, Joe Kai, Miruna David, Mara Ozolins, Louise Jackson, Zainab Abdali, Trish M. Hepburn, Frances Griffiths, Alan Montgomery, Jane Daniels, Alice Manley, Gillian Dean, Lindsay K. Armstrong-Buisseret

**Affiliations:** 1 Department of GU Medicine, University Hospitals Birmingham NHS Foundation Trust, Whittall Street Clinic, Whittall Street, Birmingham, B4 6DH UK; 2 Nottingham Clinical Trials Unit, University of Nottingham, University Park, Nottingham, NG7 2RD UK; 3 Division of Health Sciences, Warwick Medical School, University of Warwick, Coventry, CV4 7AL UK; 4 Centre for Academic Primary Care, School of Medicine, University of Nottingham, University Park, NG7 2RD UK; 5 Clinical Microbiology, University Hospitals Birmingham NHS Foundation Trust, Queen Elizabeth Hospital Birmingham, Mindelsohn Way, Edgbaston, Birmingham, B15 2GW UK; 6 Health Economics Unit, Institute of Applied Health Research, College of Medical and Dental Sciences, University of Birmingham, Edgbaston, Birmingham, B15 2TT UK; 7 Centre for Health Policy, University of the Witwatersrand, Johannesburg, South Africa; 8 Elton John Research Centre, Sussex House, 1 Abbey Road, Brighton, BN2 1ES UK

**Keywords:** Metronidazole, Lactic acid, Bacterial vaginosis, Recurrence, Sexual health, Side effects

## Abstract

**Background:**

Bacterial vaginosis is a common and distressing condition for women. Short-term antibiotic treatment is usually clinically effective, but recurrence is common. We assessed the effectiveness of intravaginal lactic acid gel versus oral metronidazole for treating recurrent bacterial vaginosis.

**Methods:**

We undertook an open-label, multicentre, parallel group, randomised controlled trial in nineteen UK sexual health clinics and a university health centre. Women aged ≥ 16 years, with current bacterial vaginosis symptoms and a preceding history of bacterial vaginosis, were randomised in a 1:1 ratio using a web-based minimisation algorithm, to 400 mg twice daily oral metronidazole tablets or 5 ml once daily intravaginal lactic acid gel, for 7 days. Masking of participants was not possible. The primary outcome was participant-reported resolution of symptoms within 2 weeks. Secondary outcomes included time to first recurrence of symptoms, number of recurrences and repeat treatments over 6 months and side effects.

**Results:**

Five hundred and eighteen participants were randomised before the trial was advised to stop recruiting by the Data Monitoring Committee. Primary outcome data were available for 79% (204/259) allocated to metronidazole and 79% (205/259) allocated to lactic acid gel. Resolution of bacterial vaginosis symptoms within 2 weeks was reported in 70% (143/204) receiving metronidazole versus 47% (97/205) receiving lactic acid gel (adjusted risk difference -23·2%; 95% confidence interval -32.3 to -14·0%). In those participants who had initial resolution and for whom 6 month data were available, 51 of 72 (71%) women in the metronidazole group and 32 of 46 women (70%) in the lactic acid gel group had recurrence of symptoms, with median times to first recurrence of 92 and 126 days, respectively. Reported side effects were more common following metronidazole than lactic acid gel (nausea 32% vs. 8%; taste changes 18% vs. 1%; diarrhoea 20% vs. 6%, respectively).

**Conclusions:**

Metronidazole was more effective than lactic acid gel for short-term resolution of bacterial vaginosis symptoms, but recurrence is common following both treatments. Lactic acid gel was associated with fewer reported side effects.

**Trial registration:**

ISRCTN14161293, prospectively registered on 18^th^ September 2017.

**Supplementary Information:**

The online version contains supplementary material available at 10.1186/s12905-023-02303-5.

## Background

Bacterial vaginosis affects 23–50% of women [1–3] and is characterised by a disruption in the vaginal microbiome associated with an offensive smelling vaginal discharge, leading to significant psychological distress and a reduction in quality of life [4]. It is associated with an increased risk of pelvic inflammatory disease, sexually transmitted infections (STIs), late miscarriage, and preterm birth [1, 2].

Current guidelines recommend the use of antibiotics, most commonly metronidazole, as first line treatment [1, 2, 5]. In those with frequent recurrences, short courses of antibiotics to treat each symptomatic episode are suggested, or regular antibiotic therapy over several weeks or months is used as prophylaxis. Antibiotic treatment leads to cure within 2–4 weeks in 51–82% of patients but recurrence is common, affecting 69–80% of women over the next 12 months [6]. The cause of treatment failure and relapse is not known but may be due to antimicrobial resistance, failure to re-establish the normal vaginal flora [7], development of a treatment resistant bacterial biofilm on the vaginal mucosa [8] and/or reinfection from a sexual partner [9]. Metronidazole treatment is associated with side effects [10] which can limit acceptability and adherence. Patients also dislike taking multiple courses of antibiotics and are concerned that they may acquire resistant bacteria [11]. Preventing and reducing antimicrobial resistance is also a public health priority through improving antibiotic stewardship including a reduction in antibiotic use [12]. The limited efficacy of current treatment for bacterial vaginosis [13] and a need to reduce antibiotic exposure highlight the need for alternative therapies. Different treatment options which have been explored include high dose metronidazole [14], antibiotic combination therapy [15], extended release clindamycin [16], agents to disrupt the bacterial biofilm [17], and probiotics [18]. At best these have provided a modest improvement in efficacy compared to standard treatment [13] and are not currently included in treatment guideline recommendations.

Lactobacilli dominate the normal vaginal flora and produce lactic acid which lowers vaginal pH inhibiting the growth of other bacterial species. Bacterial vaginosis is associated with a rise in vaginal pH and overgrowth of mostly anaerobic bacteria [1]. Intravaginal lactic acid gel can potentially be used to reduce the pH, ‘normalise’ vaginal acidity and inhibit bacterial overgrowth [19] Two recent systematic reviews have highlighted the lack of existing high quality evidence to inform the use of lactic acid gel and a need for appropriate rigorous randomised trials [20, 21].

## Methods

### Study aims and design

The metronidazole Versus lactic acId for Treating bacterial vAginosis (VITA) open-label, multicentre, parallel group, randomised controlled trial aimed to assess the effectiveness and acceptability of intravaginal lactic acid gel compared with oral metronidazole for women with recurrent bacterial vaginosis. The protocol has been previously published [22].

### Study setting and participants

Women aged ≥ 16 years with a clinical diagnosis of bacterial vaginosis based on patient reported symptoms (vaginal discharge with an offensive odour) and a history of similar symptoms on ≥ 1 occasion(s) within the past 2 years which had responded to treatment, were recruited from 19 UK sexual health centres and a university general practice health centre [22]. Microscopy confirmation of bacterial vaginosis was not required for recruitment, but local laboratory microscopy findings were recorded if undertaken as part of routine care and central laboratory microscopy assessment for bacterial vaginosis was also performed for all participants. Participants agreed to avoid vaginal douching and sexual intercourse (or use effective contraception) during treatment, and provide written informed consent. Exclusion criteria were contra-indications to either study treatment; pregnancy, breastfeeding or trying to conceive; use of other antibiotics or antifungal agents within the previous 2 weeks or planned use within 2 weeks; use of topical vaginal antibiotics, antifungals or acidifying products recently or planned within 2 weeks; previous participation in this trial; or concurrent participation in another trial involving an investigational medicinal product.

### Randomisation and masking

Delegated personnel in sites randomised participants 1:1 to lactic acid gel or metronidazole using a dedicated website maintained by Nottingham Clinical Trials Unit. Minimisation factors included: site, type of site (general practice, sexual health clinic), number of episodes of bacterial vaginosis in the previous 12 months (0, 1–3, > 3), and history of a female sexual partner in the previous 12 months (yes/no).

Study treatment was provided from standard clinic stock or via a routine prescription. Any licensed brand of metronidazole or lactic acid gel could be used and was determined by local practice or availability. Participants took metronidazole tablets 400 mg orally twice daily for 7 days, or inserted 5 ml of 4.5% lactic acid gel into the vagina before bedtime each day for 7 days. Participants were advised to start treatment on the day of receipt. If they were menstruating, those assigned lactic acid gel were advised to delay starting treatment until menstruation had finished. Due to the different routes of administration, it was not possible to blind participants and clinicians to the randomised allocation. However, the trial team excluding members of the data management/IT team remained blinded to treatment allocation until the database was locked for the final analysis.

### Outcome measures

As we are not aware of any core outcome sets for bacterial vaginosis, the primary outcome measure, collected via web-based questionnaire, was participant reported resolution of bacterial vaginosis symptoms by 2 weeks after randomisation. This reflects clinical practice where diagnosis and management is often based on history and examination. Microbiological resolution of bacterial vaginosis was also evaluated as a secondary endpoint at 2 weeks but not at the follow up assessments at 3 or 6 months. The following secondary outcomes were also captured using web based questionnaires at 2 weeks, 3 months and 6 months: adherence to treatment (2 weeks only), time to reported resolution of bacterial vaginosis symptoms, number of reported bacterial vaginosis episodes and treatment courses and time to first recurrence of symptoms.

Those not providing Week 2 and 6-month questionnaire data were contacted by phone to collect the primary outcome and details of symptom recurrence.

At baseline and Week 2, participants provided self-taken vaginal samples for microbiological assessment of bacterial vaginosis (microscopy of a gram stained vaginal smear using the Ison-Hay scoring system [23]) and STI screening (nucleic acid amplification tests for chlamydia, gonorrhoea and trichomoniasis) which were posted to a central laboratory. The secondary outcome of microbiological resolution was defined as having Ison-Hay grade 3 at baseline followed by Ison-Hay grade 0, 1, 2 or U at Week 2 based on the central laboratory findings. The prevalence of concurrent STIs at baseline and Week 2 were determined.

Additional results for the secondary outcomes of quality of life, tolerability, acceptability of treatment, healthcare resource usage and cost-effectiveness are reported elsewhere [24]. Serious adverse events were recorded if reported by participants.

### Statistical analysis

Assuming that 80% of participants receiving metronidazole would achieve symptom resolution [25], 1710 participants (855 in each treatment group) were required to detect a 6% increase in response rate to 86% in those receiving lactic acid gel at the 5% significance level (2-sided) with 90% power. To allow for loss to follow-up of 10%, it was planned to randomise 1900 participants. No interim analyses were planned therefore no adjustment to the significance level was required.

All analyses were performed using Stata® SE 15.1 (StataCorp LP, College Station, TX, USA) according to the Statistical Analysis Plan (SAP) which was finalised without knowledge of treatment allocation and prior to database lock.

The primary approach to between-group comparative analysis included all participants who were randomised and analysed according to randomised treatment group without imputation of missing outcome data. Pre-specified sensitivity analyses of the primary outcome were conducted to investigate the impact of missing data, additional baseline variables and adherence to allocated treatment.

Evaluation of the primary outcome planned to use a mixed-effects model for binary outcome including minimisation factors and whether the participant had performed vaginal douching in the 3 months prior to randomisation. However, due to model non-convergence, a generalised estimating equation for binary outcomes was applied including minimisation factors (excluding type of site since only one general practice recruited participants), and site as a panel variable and excluding whether the participant had performed vaginal douching. The adjusted odds ratio was presented in addition to the planned adjusted risk difference and adjusted risk ratio to allow for consistency in presentation with the sensitivity analyses which used a random effects logit model instead of the planned mixed-effects model, due to non-convergence issues. These changes to the proposed models were documented in the SAP.

Most secondary outcomes were analysed using appropriate regression models including minimisation factors (except site type) and outcome baseline value where measured. Side effect data were summarised using counts and proportions, according to the treatment the participant actually received, irrespective of allocation.

Pre-planned exploratory sub-group analyses were conducted to investigate whether there was an interaction affecting the primary outcome between treatment group and whether bacterial vaginosis had been confirmed by positive microscopy at baseline, presence of a concomitant STI, number of episodes of bacterial vaginosis in the 12 months before randomisation and total time with bacterial vaginosis in the 12 months before randomisation. These latter two sub-groups were inadvertently not stated in the published protocol but were included in the SAP.

The study and its reporting adhered to all CONSORT guidelines.

## Results

Recruitment occurred between 31^st^ October 2017 and 28^th^ June 2019 when the trial was closed to recruitment based on a recommendation from the Data Monitoring Committee that that the primary research question had been answered. There were no concerns around safety of the drugs or wellbeing of the participants. Follow-up of ongoing participants continued for 6 months (completed 26^th^ February 2020).

**Fig. 1:**
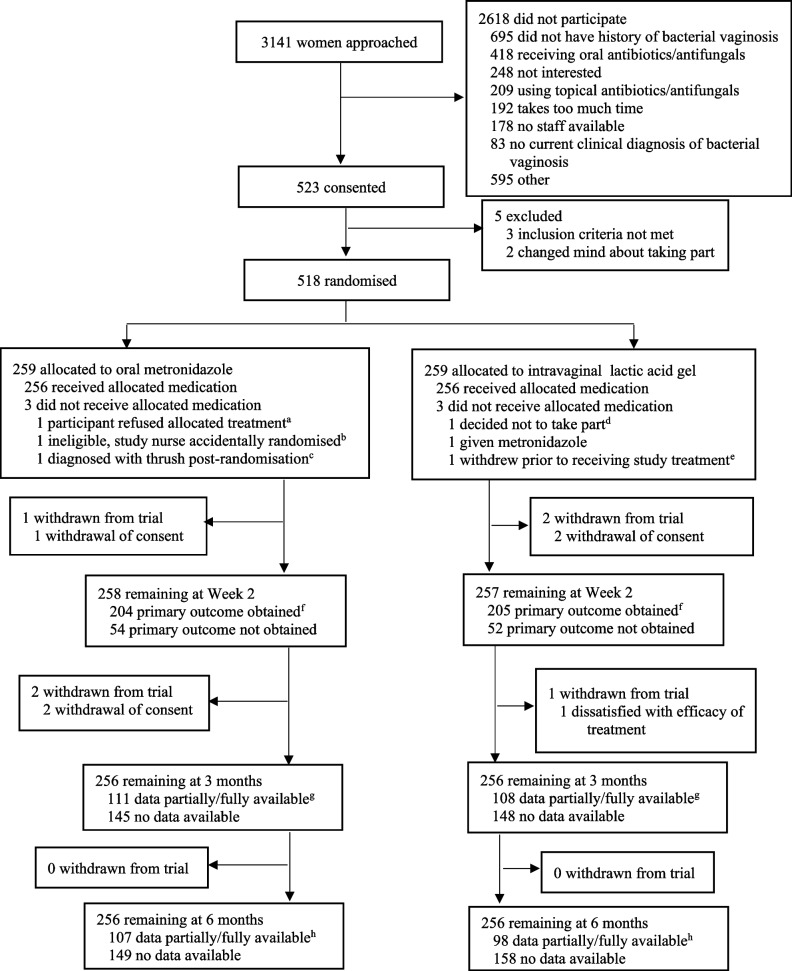
Flowchart of the VITA trial. ^a^ Prescribed lactic acid gel as refused allocated study treatment.^b^ Ineligible as taking warfarin, prescribed lactic acid gel instead.^c^ No study treatment given as participant received medication to treat thrush.^d^ Preferred metronidazole after being randomised to lactic acid gel.^e^ Included as one of the two withdrawn before Week 2 in the next box down.^f^ Includes outcomes obtained from Week 2 questionnaire where a date of resolution was given without an answer to the ‘Have your bacterial vaginosis symptoms cleared’ question, and primary outcome collected by phone. Also includes outcomes obtained from 3 month questionnaire asking about resolution by Week 2.^g^ At least one data item entered on questionnaire. ^h^ At least one data item entered on questionnaire or obtained by telephone

In total, 518 women were randomised (259 to metronidazole and 259 to lactic acid gel; Fig. 1). Baseline characteristics were similar between the two treatment groups (Table 1) with 198 (38%) participants having experienced > 3 episodes of bacterial vaginosis in the previous 12 months. Bacterial vaginosis was confirmed via microscopy of vaginal smears at baseline in 436 (84%) participants by local laboratories and in 266 (51%) by the central laboratory. Participant reported adherence to study treatment was high (Table 2).

**Table 1:** Baseline characteristics

**Characteristic**	**Oral metronidazole (*n* = 259)**	**Intravaginal lactic acid gel (*n* = 259)**	**Total (*n* = 518)**
**Age at randomisation (years)**			
Median [IQR]	27 [23–34]	27 [23–34]	27 [23–34]
**Ethnicity**^**a**^			
White	125 (48%)	126 (49%)	251 (48%)
Black Caribbean	62 (24%)	57 (22%)	119 (23%)
Mixed Race	24 (9%)	27 (10%)	51 (10%)
Black African	26 (10%)	15 (6%)	41 (8%)
Indian, Pakistani or Bangladeshi	7 (3%)	9 (3%)	16 (3%)
Other	15 (6%)	25 (10%)	40 (8%)
**Vaginal douching in the past 3 months**^**a**^			
Yes	36 (14%)	25 (10%)	61 (12%)
No	223 (86%)	233 (90%)	456 (88%)
**Frequency of douching per month**			
0–2	9 (25%)	6 (24%)	15 (25%)
3–4	9 (25%)	5 (20%)	14 (23%)
5–6	1 (3%)	3 (12%)	4 (7%)
≥ 7	17 (47%)	11 (44%)	28 (46%)
**Current use of oral contraceptive pill**^**a**^			
Combined oral contraceptive pill	27 (10%)	30 (12%)	57 (11%)
Progesterone only pill	18 (7%)	14 (5%)	32 (6%)
Contraceptive pill not currently used	214 (83%)	214 (83%)	428 (83%)
**Past history of bacterial vaginosis**			
**Approximate age when bacterial vaginosis first occurred (years)**^**b**^			
Median [IQR]	22 [18–27]	22 [19–27]	22 [19–27]
Min, max	14, 58	11, 50	11, 58
**Number of previous episodes of bacterial vaginosis in the past 12 months**			
0	3 (1%)	3 (1%)	6 (1%)
1–3	157 (61%)	157 (61%)	314 (61%)
> 3	99 (38%)	99 (38%)	198 (38%)
**Approximate total length of time in past year with bacterial vaginosis symptoms**^**a**^			
< 2 weeks	56 (22%)	40 (15%)	96 (19%)
≥ 2 weeks and < 3 months	135 (52%)	130 (50%)	265 (51%)
≥ 3 months	68 (26%)	88 (34%)	156 (30%)
Missing	0	1 (< 0.5%)	1 (< 0.5%)
**Bacterial vaginosis confirmed at baseline visit (local laboratory)**^**a**^			
Yes	217 (84%)	219 (85%)	436 (84%)
No	31 (12%)	29 (11%)	60 (12%)
Not tested	11 (4%)	10 (4%)	21 (4%)
**Baseline sample Ison-Hay grade for bacterial vaginosis (central lab)**^**c**^			
0 (no bacteria)	1 (< 0.5%)	1 (< 0.5%)	2 (< 0.5%)
1 (normal flora)	48 (19%)	62 (24%)	110 (21%)
2 (intermediate bacterial vaginosis)	62 (24%)	61 (24%)	123 (24%)
3 (confirmed bacterial vaginosis)	138 (53%)	128 (49%)	266 (51%)
U (Gram positive cocci)	3 (1%)	1 (< 0.5%)	4 (1%)
Missing	7 (3%)	6 (2%)	13 (3%)

*IQR* interquartile range

All data are n (%) or median [IQR]

^a^Missing data for one participant randomised to Intravaginal lactic acid gel

^b^Missing data for one participant randomised to Intravaginal lactic acid gel and one participant randomised to oral metroidazole

^c^Grades 0, 1, 2 and U = negative for bacterial vaginosis, grade 3 = positive for bacterial vaginosis

**Table 2:** Summary of adherence to and use of study treatment (participant reported)

**Participant reported adherence**	**Oral metronidazole (*n* = 259)**	**Intravaginal lactic acid gel (*n* = 259)**	**Overall (*n* = 518)**
**Number returning questionnaire**	**157**	**161**	**318**
**Participant took/used any of randomised treatment**			
Yes	156 (99%)	160 (99%)	316 (99%)
No	1 (1%)	1 (1%)	2 (1%)
**Course of study treatment completed**^**a**^			
Yes	144 (92%)	147 (91%)	291 (92%)
No	13 (8%)	14 (9%)	27 (8%)
**Percentage of treatment course received**			
Mean [SD]	94% [18.4%]	95% [12.9%]	95% [15.8%]
Median [IQR]	100% [100%—100%]	100% [100%—100%]	100% [100%—100%]
**Participants receiving at least 85% of treatment course**	146 (93%)	148 (92%)	294 (92%)
**Reason if treatment course not completed**^**b**^			
Accidentally missed	10 (48%)	7 (58%)	17 (52%)
Did not like using/taking it	1 (5%)	1 (8%)	2 (6%)
Side-effects of treatment	4 (19%)	0	4 (12%)
Other^c^	6 (29%)	4 (33%)	10 (30%)
**Ease of taking study treatment**			
Very easy	63 (40%)	81 (50%)	144 (45%)
Easy	50 (32%)	61 (38%)	111 (35%)
Neither easy not difficult	35 (22%)	16 (10%)	51 (16%)
Difficult	7 (4%)	2 (1%)	9 (3%)
Very difficult	1 (1%)	0	1 (< 0.5%)
Missing	1 (1%)	1 (1%)	2 (1%)
**Time from randomisation to treatment start (days)**^**d**^			
Median [IQR]	0 [0—1]	0 [0—1]	0 [0—1]
Min, max	0, 26	0, 17	0, 26
n	156	156	312
**Brand of lactic acid gel used**			
Balance Activ*®* (lactic acid 4.5%)	N/A	67 (42%)	
Relactagel*®* (lactic acid 4.5%)	N/A	90 (56%)	
Brand unknown	N/A	1 (1%)	
Missing	N/A	3 (2%)	

*IQR* interquartile range, *SD* standard deviation

All data are n (%), mean [SD] or median [IQR]

^a^As reported by the participant. Response was not changed even if contradicted by other data

^b^Reasons (mutually exclusive) are included even when the participant reported completing the course (8 in the metronidazole group, 2 in the lactic acid gel group). Four in the lactic acid gel group reported not completing the course but did not confirm one of the 4 available options

^c^Other reasons are given including some for which the ‘other’ reason was not given as ‘yes’. These were: misplaced treatment, started treatment late due to social engagements, misunderstood how to take treatment, was not prescribed study treatment, unknown in the metronidazole group; period started during treatment (five participants), vaginal itching and bleeding, lower abdominal pain in the lactic acid group

^d^Treatment start dates recorded by the participant as being before randomisation dates are assumed to be incorrect and are substituted by the randomisation date: 7 participants in the metronidazole group and 4 participants in the lactic acid gel group gave a treatment start date before the randomisation date. One start date was the same as the Week 2 questionnaire date although they indicated taking all doses

Overall, 409/518 (79%; 204 allocated to metronidazole and 205 allocated to lactic acid gel) participants provided primary outcome data. More participants reported resolution of bacterial vaginosis symptoms at Week 2 in the metronidazole group (143/204; 70%) compared with the lactic acid gel group (97/205; 47%). The adjusted risk difference was -23.2% (95% CI -32.3 to -14.0%), see Table 3. Sensitivity analyses supported these findings (Figure S1).

**Table 3:** Participant reported resolution of bacterial vaginosis at Week 2 – between group comparison^a^

**Resolution of bacterial vaginosis at Week 2**	**Oral metronidazole (*n* = 259)**	**Intravaginal lactic acid gel (*n* = 259)**	**Adjusted risk difference**^**a**^** (95% CI)**	**Adjusted risk ratio**^**b**^** (95% CI)**	**Adjusted odds ratio**^**b**^ **(95% CI)**
Yes	143 (70%)	97 (47%)	-23.2% (-32.3, -14.0)%	0.67 (0.57, 0.79)	0.38 (0.25, 0.57)
No	61 (30%)	108 (53%)			
Missing	55	54			

*CI* confidence interval

All data are n (%) unless otherwise indicated

^a^Difference is lactic acid minus metronidazole, and ratios are lactic acid/metronidazole

^b^Adjusted for: site, number of bacterial vaginosis episodes in 12 months before baseline (0, 1–3, > 3), female partner in 12 months before baseline (yes/no). Vaginal douching was not included as a covariate as it was omitted from the output due to a dependency between the independent variables

No treatment sub-group interactions were evident. There were too few participants with a STI at baseline to formally investigate differences (0 participants with gonorrhoea, 10 with chlamydia, and five with trichomoniasis). In each of the other pre-planned sub-group analyses, resolution rates were consistently higher in the metronidazole group compared to the lactic acid gel group (Table S1). Few participants took additional treatment within 2 weeks (22/154 and 20/158 of those with both resolution and additional treatment data in the metronidazole and lactic acid gel groups respectively) and resolution of symptoms in either group was similar in those who did and did not take additional medication (unadjusted risk difference -19.5% [95% CI -49.0 to 10.0%] versus -19.6% [95% CI -31.1 to -8.1%]).

Of those who reported symptom resolution of bacterial vaginosis symptoms, the median time to resolution was 7 days in the metronidazole group (*n* = 152; Q1, Q3 = 5, 14) and 8.5 days in the lactic acid gel group (*n* = 116; Q1, Q3 = 4, 14).

Subsequent data on recurrence was available for around half of those with initial symptom resolution by Week 2. Of these, 37/73 (51%) in the metronidazole group and 23/50 (46%) in the lactic acid gel group reported recurrence by 3 months, while 51/72 (71%) and 32/46 (70%) reported recurrence by 6 months respectively (Table S2 and Figure S2). The participants who provided recurrence data were similar to those who did not with respect to baseline characteristics (data not reported).

The number of participants whose symptoms had resolved by Week 2 and who provided data on the number of subsequent bacterial vaginosis episodes at 3 and 6 months was small (48 in the metronidazole group and 29 in the lactic acid gel group). There was little difference between treatment groups, with a median of one episode over the 6-month period (Q1, Q3 = 0, 3 in the metronidazole group and 0, 2 in the lactic acid gel group; adjusted incidence rate ratio was 0.97 [95% CI 0.56 to 1.69]). A post hoc investigation looked at the number of participants whose symptoms resolved at Week 2 and remained so for 6 months compared with those who either did not resolve or who developed symptoms within the 6 months following treatment. In total, 21/91 (23%) participants in the metronidazole group and 14/88 (16%) in the lactic acid gel group had symptom resolution which lasted for 6 months.

The median number of bacterial vaginosis treatment courses received between Week 2 and 6 months was similar between the treatment groups (1 [Q1, Q3 = 0, 3] in the metronidazole group, 1 [Q1, Q3 = 0, 2] in the lactic acid gel group), adjusted incidence rate was 1.03 (95% CI 0.53 to 2.01). The most common additional treatments taken for bacterial vaginosis were oral metronidazole and lactic acid gel. Metronidazole vaginal gel and clindamycin cream were used less frequently.

From the central laboratory analysis of samples, 138 (metronidazole group) and 128 (lactic acid gel group) participants had bacterial vaginosis confirmed on microscopy at baseline, and of those 77 (56%) and 73 (57%) respectively also had a microscopy sample available at Week 2. Microbiological resolution at Week 2 in those with bacterial vaginosis confirmed via microscopy at baseline was higher in the metronidazole group (59/77 participants; 77%) than in the lactic acid gel group (31/73 participants; 42%), adjusted risk difference was -34.3% (95% CI -49.1 to -19.5%).

There was a higher reported incidence of side effects in the metronidazole group compared to the lactic acid gel group, particularly of nausea (50/156 [32%] vs. 13/161 [8%]), taste changes (28/256 [18%] vs. 2/161 [1%]) and diarrhoea (31/156 [20%] vs. 9/161 [6%]) (Table S3). No serious adverse events were reported.

## Discussion

### Main findings

In women with recurrent bacterial vaginosis, 70% had resolution of their symptoms two weeks after treatment with metronidazole compared to 47% for those receiving lactic acid gel. Recurrence of bacterial vaginosis within 6 months, in the sub-set of those who had initial resolution, was frequent and similar after both treatments. Side effects were more common following metronidazole compared to lactic acid gel. Treatment success over 6 months was poor in both groups with only 23% of those receiving metronidazole and 16% of those using lactic acid gel having symptom resolution with no subsequent recurrences following treatment, although these estimates are based on less than half of the original trial population.

Our previously published qualitative study undertaken in a sub-set of 33 VITA trial participants [11] indicates that women dislike taking repeat courses of metronidazole to treat bacterial vaginosis and many prefer lactic acid gel, despite perceiving it to be less effective. This preference was based on a desire to apply treatment directly to the site of symptoms, the gel having an immediate soothing effect, concerns that frequent use of antibiotics would lead to resistance and the ready availability of lactic acid gel without requiring a medical consultation or prescription. The perceived greater efficacy and convenience of taking tablets compared to intravaginal therapy were benefits of metronidazole. Participants’ preferences for treatment were thus not based solely on short-term effectiveness but also related to ease of use, side effects and the possible long-term consequences of treatment.

Rates of adherence to metronidazole in the treatment of bacterial vaginosis have been reported to be 50–68% [26], but in our clinical trial setting reported adherence was high with participants reporting that both treatments were easy to take.

Entry into the VITA trial and assessment of treatment response were based on the presence or absence of self-reported bacterial vaginosis symptoms. An additional pre-planned analysis was performed on a sub-set of participants who had positive microscopic confirmation of bacterial vaginosis at baseline and further microscopy results available at Week 2. This showed a similar response rate to that seen in the primary analysis with microbiological resolution in 77% of participants receiving metronidazole versus 42% for lactic acid gel (compared to 70% and 47% respectively in those diagnosed with bacterial vaginosis based on symptoms). It is therefore possible that metronidazole has a greater effect on the vaginal microbiome (assessed on microscopy) than is suggested by our primary endpoint measure of symptom resolution. However, the clinical importance of microbiological resolution of bacterial vaginosis (compared to clinical symptom resolution) is uncertain because following a clinical response in either treatment group, no differences were detected in the frequency or timing of bacterial vaginosis recurrences over the next 6 months. Caution is, however, required in interpreting the limited data which were available over the full 6-month follow-up period since less than 40% of participants returned data for all three time-points.

We chose resolution of bacterial vaginosis symptoms as the primary outcome to maximise the trial’s relevance to patients and to reflect clinical practice where, in many settings, microscopy is not available, and clinical diagnosis and management is based on history and examination. Also, microbiological evidence of bacterial vaginosis can be present without symptoms [27] and cyclical changes in the vaginal flora (including bacterial vaginosis type flora) occur in the absence of treatment [28] which can make interpretation of microbiological cure difficult. Microscopy evaluated resolution of bacterial vaginosis at Week 2 was included as a secondary outcome in the trial and was based on the Ison and Hay criteria recommended in the UK National Guideline for the Management of Bacterial Vaginosis [1]. Diagnosis using Ison and Hay criteria correlates closely with the two other commonly used approaches to microbiologic bacterial vaginosis diagnosis, Amsel’s criteria [23] and Nugent’s score [29].

From a patient perspective, the symptoms of discharge and malodour are the main cause of physical and emotional distress associated with bacterial vaginosis, and have an impact on self-esteem and relationships, and restrict sexual activity [4, 11]. Our choice of primary endpoint and findings are therefore of direct relevance to patients and those providing treatment for bacterial vaginosis.

Individuals with frequently recurrent bacterial vaginosis respond less well to treatment [14]. However, the frequency of prior bacterial vaginosis in the VITA trial did not predict symptom resolution differently between the two treatment groups. In those successfully treated with either metronidazole or lactic acid gel the same proportion (around a third) reported having had bacterial vaginosis on more than three occasions in the past year.

### Strengths and limitations

VITA was a large trial which included an ethnically diverse patient population. The trial was pragmatic and reflected common clinical practice ensuring that our results are likely to be widely applicable.

We recognise several potential limitations. It was not possible to blind participants or clinicians to the treatment allocation which raises the possibility of reporting bias. However, we think bias is unlikely because a pre-planned secondary analysis of outcomes based on microscopy findings (performed by technicians who were blind to treatment allocation) showed a similar, if slightly larger, difference in treatment efficacy compared to the primary analysis of participant reported symptoms.

The self-reporting of patient symptoms may have overestimated the number of women with bacterial vaginosis and this may have occurred more commonly in those women in the metronidazole arm who were at greater risk of developing thrush type symptoms. However, microbiological confirmation of bacterial vaginosis at baseline occurred in 84% of participants according to local laboratory analysis (which would apply in routine practice) and 51% in a central laboratory analysis suggesting that our findings remain clinically relevant. It is likely that women with microbiological evidence of bacterial vaginosis in the absence of symptoms are at increased risk of pelvic inflammatory disease and adverse pregnancy outcomes but it is not known whether treating bacterial vaginosis in this scenario can improve outcomes and further clinical trials are required to address this.

We allowed use of any lactic acid gel dispensed in 5 ml applicators, with the decision regarding the actual brand delegated to the investigator. Participants reported use of just two brands, which both contained 4.5% lactic acid balanced to ph3.8 and the same excipients which were both commercially available. It is therefore highly likely that the effectiveness of these products were similar. Theoretically the effectiveness could differ if factors such as the lactic acid isomer ratio [30] or osmolality [31] varied between brands, but these are unknown and a specific randomised trial would be needed to investigate this.

The loss to follow-up rate for collection of the primary endpoint was 21% compared to a predicted rate of 10% [22]. Sensitivity analyses were performed including assumptions that participants in both groups with missing data had symptom resolution, or that they did not have symptom resolution. In both scenarios the adjusted risk difference was similar (-18% compared to the primary analysis risk difference of -23%). However, it remains possible that the reasons for loss to follow up could have differed between the two treatment groups.

### Comparison to other studies

Four previous randomised controlled trials have evaluated lactic acid gel for the treatment of bacterial vaginosis with cure reported in 23–100% of women [32–35]. These studies were small (*n* = 31 to *n* = 125) and had a significant risk of bias [20]. Consistent with our results, two studies found lactic acid to be inferior to metronidazole, although one study suggested equivalency. Differences in lactic acid gel formulation, bacterial vaginosis diagnostic criteria and timing of assessments were not consistent between studies and may partially explain the observed differences in reported efficacy.

### Interpretation

The VITA trial provides robust measures of treatment response and side effects for metronidazole and lactic acid gel 2 weeks after treatment with additional, more limited, data over a 6-month time period. In the absence of an effective long-term treatment for recurrent bacterial vaginosis, these findings will help women with bacterial vaginosis and their clinicians make informed decisions about therapy, taking account of women’s individual preferences.

The VITA trial indicates that there is a higher initial clinical response to metronidazole than lactic acid gel but that recurrence of bacterial vaginosis following either treatment is common. For some women, oral metronidazole may be favoured because of its higher short-term efficacy. However, others may prefer intravaginal lactic acid gel if ease of use, avoiding antibiotic side effects and resistance, or access to treatment without medical prescription are of greater importance.

## Conclusion

Oral metronidazole has greater short-term efficacy in the treatment of recurrent bacterial vaginosis than intravaginal lactic acid gel, however women’s preference for therapy is influenced not just by efficacy but also by ease of use and a desire to avoid antibiotics if possible. Current treatments for recurrent bacterial vaginosis have limited efficacy and further research into pathogenesis and management, preferably limiting the use of antibiotics, is required.

## Supplementary Information

**Additional file 1: Figure S1.** Forest plot for primary outcome and sensitivity analyses.

**Additional file 2: Figure S2.** Kaplan Meier plot for recurrence of bacterial vaginosis symptoms against time.

**Additional file 3: Table S1.** Participant reported resolution of bacterial vaginosis symptoms at Week 2 – between-group comparison by sub-group.

**Additional file 4: Table S2.** Time to first recurrence of bacterial vaginosisfor those whose symptoms resolved within 2 weeks.

**Additional file 5: Table S3.** Summary of side effects reported on Week 2 questionnaire.

## Data Availability

The datasets containing individual participant data analysed during the VITA trial will be available upon request from the NCTU (ctu@nottingham.ac.uk) a minimum of 6 months after publication of this main results paper. Access to the data will be subject to review of a data sharing and use request by a committee including the Chief Investigator and Sponsor and will only be granted upon receipt of a data sharing and use agreement. Any data shared will be pseudo-anonymised which may impact on the reproducibility of published analyses. The protocol and statistical analysis plan are freely available on the NIHR Journals Library website: https://fundingawards.nihr.ac.uk/award/15/110/02.

## Abbreviations

NCTU: Nottingham Clinical Trials Unit
Q1: 25^Th^ centile
Q3: 75^Th^ centile
SAP: Statistical Analysis Plan
STI: Sexually transmitted infection
UK: United Kingdom
VITA: Metronidazole Versus lactic acid for Treating bacterial vAginosis
