# Supplementary material for: Intravaginal lactic acid gel versus oral metronidazole for treating women with recurrent bacterial vaginosis: the VITA randomised controlled trial
Source: BMC Womens Health. 2023 May 9;23:241. doi: 10.1186/s12905-023-02303-5 (PMC10169495; doi:10.1186/s12905-023-02303-5)
Supplement: Supplementary file 1 — Additional file 1: Figure S1. Forest plot for primary outcome and sensitivity analyses. [file 12905_2023_2303_MOESM1_ESM.docx]

**Figure S1: Forest plot for primary outcome and sensitivity analyses**

*CI*, confidence interval

Multiple imputation: No missing covariate data. Overall resolution is the only imputed variable, using chained equations and augment option owing to prediction problems. Estimation uses a general linear model for binary outcome.

Exclude if not received allocated treatment: Participants excluded from the analysis if they had not received any of their allocated treatment.

Further baseline variable adjustment: Vaginal douching, ethnicity and time with bacterial vaginosis added to the original model

Assuming missing data not resolved: All participants who had missing primary outcome data were assumed not to have resolved.

Assuming missing data resolved: All participants who had missing primary outcome data were assumed to have resolved.

Include as treatment received: Participants were included in the treatment group for the treatment they actually received irrespective of randomisation.
