# Supplementary material for: Intravaginal lactic acid gel versus oral metronidazole for treating women with recurrent bacterial vaginosis: the VITA randomised controlled trial
Source: BMC Womens Health. 2023 May 9;23:241. doi: 10.1186/s12905-023-02303-5 (PMC10169495; doi:10.1186/s12905-023-02303-5)
Supplement: Supplementary file 2 — Additional file 2: Figure S2. Kaplan Meier plot for recurrence of bacterial vaginosis symptoms against time. [file 12905_2023_2303_MOESM2_ESM.docx]

**Figure S2: Kaplan Meier plot** **for recurrence of bacterial vaginosis symptoms over time for participants with initial resolution of symptoms**
