# Supplementary material for: Intravaginal lactic acid gel versus oral metronidazole for treating women with recurrent bacterial vaginosis: the VITA randomised controlled trial
Source: BMC Womens Health. 2023 May 9;23:241. doi: 10.1186/s12905-023-02303-5 (PMC10169495; doi:10.1186/s12905-023-02303-5)
Supplement: Supplementary file 3 — Additional file 3: Table S1. Participant reported resolution of bacterial vaginosis symptoms at Week 2 – between-group comparison by sub-group. [file 12905_2023_2303_MOESM3_ESM.docx]

**Table S1: Participant reported resolution of bacterial vaginosis symptoms at Week 2 – between-group comparison by sub-group**

| **Sub-group** | **Metronidazole vs lactic acid gel** | **Treatment difference**  **(95% CI)^a^** | **Estimate of treatment-sub-group interaction**  **(95% CI)^b^** |
| --- | --- | --- | --- |
| **Presence of concomitant STI at baseline^c^** |  |  |  |
| No (n=259) | 67% (85/126) vs 50% (66/133) | -17.8% (-29.6, -6.0)% | Insufficient data available |
| Yes (n=8)^d^ | 100% (5/5) vs 0% (0/3) | -100% (not calculable) |  |
| **Bacterial vaginosis confirmed by positive baseline microscopy (central laboratory Ison-Hay grade 3)^e^** |  |  |  |
| No (n=190) | 65% (58/89) vs 46% (46/101) | -19.6% (-33.5, -5.8)% |  |
| Yes (n=212) | 75% (83/111) vs 49% (49/101) | -26.3 (-38.9, -13.6)% | 2.1% (-11.6, 15.7)%  p = 0.77 |
| **Number of episodes of bacterial vaginosis in 12 months before baseline** |  |  |  |
| 0 (n=4) | 100% (1/1) vs 67% (2/3) | -33.3% (-86.7, 20.0)% |  |
| 1-3 (n=242) | 75% (91/122) vs 51% (61/120) | -23.8% (-35.6, -11.9)% | -2.0% (-21.2, 17.0)%  p = 0.83 |
| >3 (n=163) | 63% (51/81) vs 42% (34/82) | -21.5% (-36.5, -6.5)% |  |
| **Total time with bacterial vaginosis in 12 months before baseline** |  |  |  |
| <2 weeks (n=78) | 72% (31/43) vs 57% (20/35) | -15.0% (-36.1, 6.2)% |  |
| ≥2 weeks and <3 months (n=209) | 73% (78/107) vs 53% (54/102) | -20.0% (-32.8, -7.1)% | -3.1% (-27.8, 21.6)%  p = 0.81 |
| ≥3 months (n=122) | 63% (34/54) vs 34% (23/68) | -29.1% (-46.2, -12.0)% | -11.1% (-38.5, 16.4)%  p = 0.43 |

*CI*, confidence interval

Data are n (%) unless otherwise indicated.

^a^ Simple treatment differences are presented, due to non-convergence of more complicated models.

^b^ Adjusted for number of female partners in last 12 months, and number of episodes in last 12 months.

^c^ Chlamydia, gonorrhoea or trichomoniasis in central laboratory sample. Two samples had an indeterminate result for all three STIs, and a third had a mixture of indeterminate and negative results; all three samples are therefore missing for overall STI.

^d^ All in metronidazole group resolved; none resolved in lactic acid gel group.

^e^ The baseline covariate is dependent on other variables in the analysis.
