# Supplementary material for: Intravaginal lactic acid gel versus oral metronidazole for treating women with recurrent bacterial vaginosis: the VITA randomised controlled trial
Source: BMC Womens Health. 2023 May 9;23:241. doi: 10.1186/s12905-023-02303-5 (PMC10169495; doi:10.1186/s12905-023-02303-5)
Supplement: Supplementary file 4 — Additional file 4: Table S2. Time to first recurrence of bacterial vaginosisfor those whose symptoms resolved within 2 weeks. [file 12905_2023_2303_MOESM4_ESM.docx]

**Table S2: Time to first recurrence of bacterial vaginosis (days) for those whose symptoms resolved within 2 weeks (participant reported)**

| **Resolution/recurrence of bacterial vaginosis** | **Oral metronidazole**  **(n =259)** | **Intravaginal lactic acid gel**  **(n =259)** |
| --- | --- | --- |
| **Resolved by Week 2** | 143 | 97 |
| **Recurred within 3 months** | 37/ 73 (51%) | 23/ 50 (46%) |
| **Recurred within 6 months** | 51/72 (71%) | 32/46 (70%) |
| **Including censored times** |  |  |
| Number with time to recurrence | 73 | 50 |
| Median time to recurrence (IQR) | 92 (49, .)^a^ | 124 (63, .)^a^ |
| **Without censored times** |  |  |
| Number with time to recurrence | 43 | 25 |
| Median time to recurrence (IQR) | 54 (33-80) | 66 (25-79) |

*IQR*, interquartile range

^a^ It was only possible to calculate the 25% quartile as there were not enough uncensored ‘events’.

Time is censored for those without recurrence at the latest time for which data up to that point are available, and is calculated from the date of resolution. If a participant had no recurrence up to 3 months, and 6 month data were missing, then overall (6 month) recurrence status would also be missing, and time to recurrence is censored at 3 months. Time to recurrence is censored at 6 months if data are available at both 3 and 6 months, but no recurrence was reported.

Denominators are greater for median times than for 6 month recurrence data due to times censored at 3 months.
