# Supplementary material for: Intravaginal lactic acid gel versus oral metronidazole for treating women with recurrent bacterial vaginosis: the VITA randomised controlled trial
Source: BMC Womens Health. 2023 May 9;23:241. doi: 10.1186/s12905-023-02303-5 (PMC10169495; doi:10.1186/s12905-023-02303-5)
Supplement: Supplementary file 5 — Additional file 5: Table S3. Summary of side effects reported on Week 2 questionnaire. [file 12905_2023_2303_MOESM5_ESM.docx]

**Table S3: Summary of side effects reported on Week 2 questionnaire**

|  | **Oral metronidazole (n=258)** | **Intravaginal lactic acid gel (n=258)** |
| --- | --- | --- |
| Number of questionnaires expected | 256 | 258 |
| Number of questionnaires returned | 156 | 161 |
| Number of participants experiencing one or more episodes of |  |  |
| Nausea | 50 (32%) | 13 (8%) |
| Vomiting | 9 (6%) | 2 (1%) |
| Taste changes | 28 (18%) | 2 (1%) |
| Vaginal irritation | 44 (28%) | 34 (21%) |
| Abdominal pain | 31 (20%) | 27 (17%) |
| Diarrhoea | 31 (20%) | 9 (6%) |

All data are n (%).

Tabulated by treatment received. Two participants from each treatment group received the other study treatment; one participant allocated to lactic acid received no study treatment and withdrew before any follow-up; one participant allocated to metronidazole received a non-study treatment but provided no side effect data and hence is not included in this table.
